# Revascularization and outcomes in Veterans with moderate to severe ischemia on myocardial perfusion imaging

**DOI:** 10.1186/s40779-017-0121-x

**Published:** 2017-04-01

**Authors:** David E. Winchester, Alexander J. Bolanos, Anita Wokhlu, Rebecca J. Beyth, Leslee J. Shaw

**Affiliations:** 1 Malcom Randall VAMC, North Florida/South Georgia Veterans Health System, 1601 SW Archer Rd, Box 111-D, Gainesville, FL USA; 2 Division of Cardiovascular Medicine, University of Florida College of Medicine, Malcom Randall VAMC, 1601 SW Archer Rd, Box 111-D, Gainesville, FL USA; 3 Department of Medicine, University of Florida College of Medicine, Gainesville, FL USA; 4 Division of General Internal Medicine, University of Florida College of Medicine, Gainesville, FL USA; 5 Division of Cardiology, Emory University School of Medicine, Atlanta, GA USA

**Keywords:** Myocardial ischemia, Nuclear myocardial perfusion imaging, Veterans, Revascularization

## Abstract

**Background:**

The prevalence of ischemia on nuclear myocardial perfusion imaging (MPI) has been decreasing. Recent research has questioned the benefit of invasive revascularization for patients with moderate to severe ischemia. We hypothesized that patients with moderate to severe ischemia could routinely undergo successful revascularization.

**Methods:**

We analyzed data from 544 patients who underwent an MPI at a single academic Veterans Affairs Medical Center. Patients with moderate to severe ischemia, defined as a summed difference score (SDS) 8 or greater, were compared to the rest of the cohort.

**Results:**

Of the total cohort (*n* = 544), 39 patients had MPI studies with resultant moderate to severe ischemia. Patients with ischemia were more likely to develop coronary artery disease (74.4% versus 38.8%, *P* < 0.0001) and have successful revascularization (38.5% versus 4.0%, *P* < 0.0001) during the following year. Revascularization was attempted in 31 patients with moderate to severe ischemia, though only 15 (47%) of these attempts were successful. Ischemia was predictive of myocardial infarction (5.1% versus 0.8%, *P* = 0.01) within 1 year.

**Conclusion:**

Moderate to severe ischemia is an uncommon finding in a contemporary nuclear laboratory. Among patients with ischemia, revascularization is typically attempted but is frequently unsuccessful.

**Trial registration:**

This trial does not appear on a registry as it is neither randomized nor prospective.

## Background

Myocardial perfusion imaging (MPI), which detects myocardial ischemia, is reliable at detecting obstructive coronary artery disease (CAD) [1]. When the MPI is normal, patients are at a lower risk for cardiovascular events, usually for at least 1 year following the test [2]. When MPI demonstrates a large burden of myocardial ischemia, cohort evidence suggests that revascularization is superior to medical therapy for reducing cardiovascular events [3]. As such, MPI is commonly used to decide in which patients invasive revascularization should be pursued.

In contrast, randomized clinical trial evidence suggests that revascularization for stable CAD is not effective at reducing cardiovascular events [4, 5]. This variation of results in the literature has led to a degree of clinical equipoise regarding the management of abnormal stress testing, while funding for the International Study of Comparative Health Effectiveness with Medical and Invasive Approaches (ISCHEMIA) trial is currently underway [6, 7]. In the absence of a clear clinical benefit, patients and physicians may depend on other factors to make clinical management decisions. Percutaneous revascularization success has improved significantly since its inception [8]. Patient choice and clinical factors, such as renal disease and bleeding risk, may be barriers to the use of a revascularization strategy.

To better understand decisions about the management of abnormal MPI and patterns of revascularization, we conducted this investigation in a population of patients with moderate to severe myocardial ischemia at a large Veterans Affairs Medical Center. We hypothesized that revascularization would be the predominant strategy and that the presence of ischemia would be predictive of future cardiovascular events.

## Methods

### Study design

We conducted a retrospective cohort study of patients at a single academically affiliated Veterans Affairs Medical Center who underwent MPI between December 2010 and July 2011. The study protocol was reviewed by our Institutional Review Board, which waived the requirement for informed consent. Two cohorts were defined: 1) patients with moderate or severe ischemia and 2) patients with mild or no ischemia. Data for the subjects were retrieved from the Veterans Affairs Computerized Patient Record System and included demographics, baseline clinical characteristics, and the results from their MPI. MPI results, including the summed stress score, summed rest score, summed difference score (SDS), and the final interpretation of the MPI (e.g., normal or abnormal) were obtained. We defined an SDS of 8 or greater as predictive of moderate to severe ischemia.

MPI was conducted as either technetium-^99^m single photon emission computed tomography combined with either treadmill exercise stress or regadenoson vasodilation or as rubidium-82 positron emission tomography with regadenoson. The MPI results were interpreted by an interdisciplinary team that included faculty from nuclear medicine, cardiology, and radiology. Reporting standards for MPI were followed [9]. Any physician or provider at our facility had the authority to order an MPI, regardless of specialty.

### Statistical analysis

The primary outcome of this study was to determine if subjects with moderate to severe ischemia were more likely to have a successful revascularization within 1 year after an MPI than those with mild/no ischemia. We compared outcomes using Chi-square tests. Baseline variables were compared using the Mann-Whitney *U* tests and chi-squares as appropriate. The secondary outcome was to compare the rates of myocardial infarction (MI), between the two cohorts at 1 year. Data were analyzed using SPSS version 21 (IBM, Armonk, NY). A *P*-value of <0.05 was predefined as a significant difference. The Strengthening the Reporting of Observational Studies in Epidemiology method was used in the development of this investigation [10].

## Results

### Clinical characteristics

The study population was predominantly male, which is typical of a Veteran population, and the median age was 63. Patients with no to mild ischemia (SDS < 8) were 64 (61–70) years, and patients with moderate to severe ischemia (SDS ≥ 8) were 63 (58–67) years (*P* = 0.07), there has no significant difference between the two groups. Clinical characteristics of the 544 veterans are summarized in Table 1. Most baseline clinical variables were not different between the two groups, but the moderate to severe ischemia cohort was more likely to have CAD or an abnormal ECG at baseline. Symptom burden (i.e., chest pain or dyspnea) was similar between the groups. Of the total population, 39 (7.1%) had an SDS score of 8 or greater, and the median SDS was 11.

**Table 1:** Baseline demographic and clinical characteristics

Item	SDS ≥ 8 (*n* = 39)	SDS < 8 (*n* = 505)	*P* value
Age (year)	63(58–67)	64(61–70)	0.07
Clinical Characteristics [*n*(%)]			
Chest pain	20(51.3)	248(49.1)	0.79
Dyspnea	15(38.5)	208(41.2)	0.74
CAD	29(74.4)	196(38.8)	<0.0001
DM	17(43.6)	209(41.4)	0.79
HTN	30(76.9)	418(82.8)	0.36
HLD	34(87.2)	381(75.4)	0.10
Tobacco use	144(28.5)	8(20.5)	0.15
Obesity	21(53.8)	345(68.5)	0.06
Abnormal ECG	28(71.8)	266(52.7)	0.02
Outcomes [*n*(%)]			
Coronary angiography	31(79.5)	48(9.5)	<0.0001
Successful revascularization	15(38.5)	20(4.0)	<0.0001
MI within 1 year	2(5.1)	4(0.8)	0.01

*SDS* Summed difference score, *CAD* Coronary artery disease, *DM* Diabetes mellitus, *HTN* Hypertension, *HLD* Hyperlipidemia, *ECG* Electrocardiogram, *MI* Myocardial infarction. Age is expressed as interquartile range (*IQR*)

### Outcomes

**Fig. 1:**
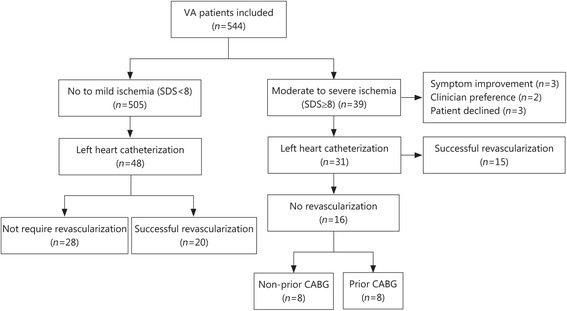
Patient flow diagram. Flowchart demonstrating the distribution of the two cohorts of patients and their clinical management after myocardial perfusion imaging (MPI). SDS: Summed difference score, CABG: Coronary artery bypass graft, VA: Veterans affairs

Patients with moderate to severe ischemia were more likely to undergo coronary angiography (79.5% versus 9.5%, *P* < 0.0001) and successful revascularization (38.5% vs. 4.0%, *P* < 0.0001, Table 1). Figure 1 demonstrates the flow of patients through post-MPI management. Eight patients in the moderate to severe cohort did not undergo left heart catheterization due to the improvement of symptoms (*n* = 3), the clinician’s preference (*n* = 2), or the patient declining angiography (*n* = 3). Of those in the moderate to severe ischemia population who underwent angiography, over half did not have a successful revascularization (*n* = 16). In nearly all these 16 patients, their coronary anatomy and disease process were not suitable for mechanical revascularization due to chronic total occlusion of the vessel or the anatomy from prior coronary artery bypass grafting (CABG) surgery that could not be addressed percutaneously (Table 2). In only one case was PCI attempted and failed, in all the others PCI was not attempted. A small proportion of patients suffered an MI (*n* = 6) within one year of the MPI, and that was more frequent in the moderate to severe cohort (5.1% versus 0.8% in the no to mild ischemia cohort, *P* = 0.01, Table 1).

**Table 2:** Details of patients with ischemia who underwent angiography without successful revascularization (*n* = 16)

No. of patient	SDS	Attempted revascularization	Reason for no revascularization
1	8	None	Poor targets, CTO
2	8	None	Poor targets, CTO
3	9	None	Poor targets, CTO
4	9	None	Poor targets, non-obstructive CAD
5	9	None	Poor targets, prior CABG
6	9	None	Poor targets, prior CABG
7	10	None	Poor targets, CTO
8	10	PCI	Failed PCI, prior CABG
9	11	None	Poor targets, prior CABG
10	12	None	Poor targets, native disease
11	12	None	Poor targets, prior CABG
12	14	None	Poor targets, CTO
13	16	None	Poor targets, prior CABG
14	16	None	Poor targets, native disease
15	21	None	Poor targets, prior CABG
16	25	None	Poor targets, prior CABG

*CABG* Coronary artery bypass grafting, *CAD* Coronary artery disease, *CTO* Chronic total occlusion, *PCI* Percutaneous coronary intervention, *SDS* Summed difference score

## Discussion

In this investigation, we demonstrated that moderate to severe ischemia is uncommon in a contemporary nuclear cardiology laboratory. This finding is consistent with a larger cohort spanning almost two decades, which showed the prevalence of decreasing ischemia from 29.6% in 1991 to 5.0% in 2009 [11]. The relative scarcity of notable ischemia introduces uncertainty regarding the utility of widespread MPI testing. Professional societies and consumer groups have addressed these questions through the development of the Appropriate Use Criteria and the Choosing Wisely campaign [12, 13].

We observed that patients with moderate to severe ischemia are more likely to undergo angiography than patients with mild/no ischemia. More importantly, we observed that in this Veteran population with a high prevalence of CAD, revascularization that was attempted was frequently unsuccessful. We also observed reasons why revascularization was not pursued for some patients.

As previously noted, cohort data have suggested that revascularization is superior to medical management in patients with moderate to severe myocardial ischemia [3]. Thus, our findings of the greater use of coronary angiography in this cohort were not surprising. The notable finding from our investigation was that despite this strong clinical preference, nearly half of this cohort was not able to be revascularized. A variety of clinical variables contribute to failure of coronary revascularization, which include vessel tortuosity, plaque calcification, and lesion location. Stenting within bypass grafts can be challenging, and sometimes ischemia is related to a chronic total vessel occlusion. When a low procedural success rate is added to the costs and risks associated with coronary angiography and revascularization, it may be reasonable to first attempt to manage patients conservatively with medical therapy, but these decisions need to be made based on individualized patient care. Ample evidence also suggests that cardiovascular risk factors such as smoking, blood pressure, diet, and exercise are undertreated and are more effective at reducing cardiovascular events.

We observed that even without a concerted effort at medical therapy, some patients’ symptom profiles improved after the MPI and no longer warranted revascularization. Despite the presence of ischemia, both physicians and patients in our cohort found reasons to decline coronary angiography. Those that declined angiography were in the minority, and the opportunity to improve decision-making likely exists. A survey of patients and cardiologists found widespread misunderstanding of the benefits of revascularization among patients, and although cardiologists demonstrated better understanding of the benefits of revascularization, a substantial portion reported that they would perform revascularization even in situations where they recognized that there was no clinical benefit [14]. A wide variety of medical therapies are available to reduce the symptoms of angina, and shared decision-making tools are available for guiding patients and physicians through revascularization options [15, 16].

A medical-therapy-first approach must be considered in the context of the prognostic implications of moderate to severe ischemia on an MPI. As with prior reports, we observed a higher rate of MI at 1 year after an MPI was performed within the moderate to severe cohort. While the COURAGE trial did not show revascularization to be superior at reducing cardiovascular events, a sub-study of patients with serial MPIs showed that revascularization was more effective than medical therapy at reducing the ischemia burden. The relationship between myocardial ischemia and the pathophysiology of an MI is complex [6]. The highly anticipated ISCHEMIA trial (www.ischemiatrial.org) should provide robust evidence on this important clinical management question.

### Limitations

This retrospective investigation was performed in a large VA health care center. The study population was predominantly male; therefore, its generalizability to women or to other non-VA academic centers is limited. As a non-randomized trial, we cannot make any distinctions about causality regarding the decisions to undergo revascularization between the study cohorts, but can report data on decisions as to why revascularization was not pursued in some patients with ischemia. Decisions to pursue revascularization need to be made on an individual basis, and our findings are intended to describe contemporary practices and not to discourage revascularization.

## Conclusion

In relation to the amount of MPI tests that were ordered within the Veteran population, moderate to severe ischemia was an uncommon finding. Among the patients with ischemia, revascularization was typically attempted and was frequently unsuccessful. Patient preferences and therapy goals are important considerations for revascularization and should ideally be addressed even before an MPI is pursued.

## Abbreviations

CABG: Coronary artery bypass grafting
CAD: Coronary artery disease
ISCHEMIA: International study of comparative health effectiveness with medical and invasive approaches
MI: Myocardial infarction
MPI: Myocardial perfusion imaging
SDS: Summed difference score
